# Exploring the Temporal Patterns of Right Ventricular Pacing Burden

**DOI:** 10.19102/icrm.2023.14104

**Published:** 2023-10-15

**Authors:** Rahul K. Chattopadhyay, Mrinal Thakur, Rucchira Wickramasinghe, Julie Hayes, Panagiota A. Chousou, Vassilios S. Vassiliou, Peter J. Pugh

**Affiliations:** 1 Department of Cardiology, Addenbrooke’s Hospital Cambridge University Hospitals NHS Foundation Trust, Cambridge, UK; 2 Norwich Medical School University of East Anglia, Norwich, UK; 3 Department of Cardiology, Norfolk and Norwich University Hospitals NHS Foundation Trust, Norwich, UK

**Keywords:** Conduction system pacing, pacing-induced cardiomyopathy, right ventricular pacing burden

## Abstract

Elevated right ventricular pacing (RVP) burdens are associated with the development of pacing-induced cardiomyopathy. This association is alluded to in the recent European and American pacing guidelines where anticipated pacing burden forms part of the indications for conduction system pacing. Understanding the temporal pattern of RVP burden is important with respect to anticipating future burden and ensuring that the most appropriate pacing modality is selected for patients. To the best of our knowledge, this is the first study to assess how RVP burden changes over time in different pacing indications. A retrospective, single-center, observational study was performed. RVP burdens from pacing checks were extracted and plotted against 6-month time “bins.” Graphical plots of RVP burdens for different pacing indications were produced. There was no significant change in the RVP burden across time, independent of the initial pacing indication. Individuals with sinus node disease (SND) and a P–R interval of >250 ms had increased RVP burden. Other than patients with SND and a P–R interval of <250 ms, individuals had pacing burdens higher than those proposed in both the European and American pacing guidelines for conduction system pacing.

## Introduction

The deleterious impact of right ventricular pacing (RVP) burden has been well established,^1,2^ with a number of studies demonstrating the association between high RVP burden and the development of pacemaker-induced cardiomyopathy (PICM).^3–5^ Conduction system pacing is becoming increasingly established as a suitable and possibly superior alternative to conventional pacing for those considered to be at risk of PICM.^6^ This risk of developing PICM is now reflected in the latest pacing guidelines: consideration of conduction system pacing is recommended for patients with an anticipated RVP burden of >20% (European guidelines)^7^ or >40% (American guidelines).^8^

Studies on RVP burden have primarily looked at RVP at a single time point^9^ or at cumulative RVP.^10^ Given the need to anticipate elevated pacing burdens, we wish to understand the temporal pattern of RVP burden in different pacing indication subgroups in order to help facilitate identification of patients who might benefit from conduction system pacing approaches such as His-bundle pacing.

## Methods

This was a retrospective, single-center, observational study assessing pacemakers implanted during a 5-year period from May 2015 to the end of April 2020, with follow-up until March 2022. The study received approval from the institutional review board of Addenbrooke’s Hospital (PRN 9009). As the study was retrospective in nature, the need for patient consent was waived. Patients who received a first pacemaker implant for bradycardia during the study period were identified from the institution’s Cardiac Rhythm Management (CRM) database and were included. The CRM database includes the pacing indication along with each patient’s demographics (age, sex) and clinical data for all implants.

All devices included in this study were either single- or dual-chamber pacemakers implanted for a bradycardia indication. This study was prompted by the guidelines for conduction system pacing and, as such, patients undergoing cardiac resynchronization therapy or His-bundle pacing for a bradycardia indication were not included in the study.

The center has a proactive cardiac devices clinic, which follows up with patients on a 6-month basis until it is felt that the device has been optimized, at which point the follow-up interval is increased to yearly. In cases of high RVP burden, the situation is discussed with a cardiology consultant with a special interest in cardiac devices to assess whether device reprogramming may facilitate a reduction in pacing burden. At the point of implant, manufacturer-specific algorithms such as Managed Ventricular Pacing (Medtronic, Minneapolis, MN, USA) or Search AV+ (Boston Scientific, Marlborough, MA, USA) are utilized.

A detailed examination of the electronic record at the time of pacemaker implantation was performed to identify if the arrhythmia requiring pacing was continuous or intermittent.

RVP burden was identified on subsequent pacemaker checks and placed into 6-month time “bins” to facilitate analysis. In cases where multiple checks were performed during a single 6-month period, the mean value was taken.

## Results

### Baseline characteristics

A total of 1263 patients underwent pacing for a bradycardia indication during the aforementioned 5-year period. They had a mean age of 78.5 (standard deviation, 10.75) years, and 801 patients (63%) were men **(Table 1)**. The median follow-up was 826 days (interquartile range, 458.8–1215.3 days). A total of 1100 patients had at least one follow-up pacing check after the initial 6-week check. Of the 1263 devices, 198 (15.7%) were single-chamber devices, with the remaining being dual-chamber devices. All of the devices were either from Medtronic or Boston Scientific.

### Temporal pattern of right ventricular pacing

**Figure 1:**
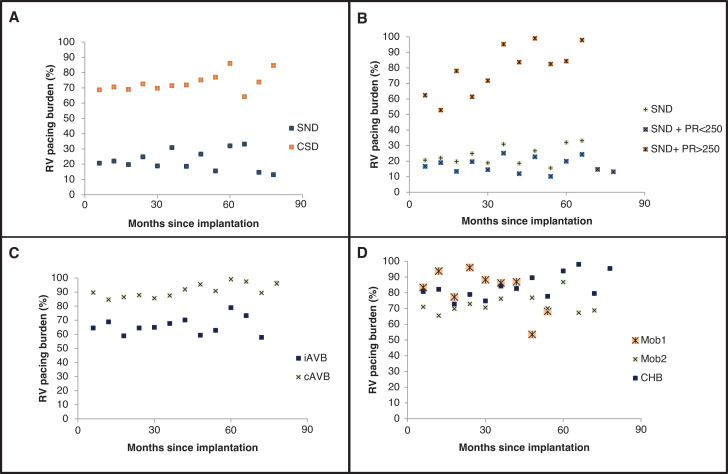
Differences in right ventricular pacing burden between different patient groups according to device indication. **A:** Sinus node disease versus conduction system disease. **B:** Sinus node disease stratified by P–R interval. **C:** Atrioventricular block stratified by intermittent versus continuous block. **D:** Different atrioventricular block manifestations. *Abbreviations:* cAVB, continuous atrioventricular block; CHB, complete heart block; CSD, conduction system disease; iAVB, intermittent atrioventricular block; RV, right ventricular; SND, sinus node disease.

**Figure 1A** plots the relationship of RVP burden with time for sinus node disease (SND) compared to conduction system disease. **Figure 1B** shows the impact of P–R prolongation on RVP burden in patients with SND. **Figure 1C** considers the impact of intermittent block compared to continuous block in patients with atrioventricular (AV) block. **Figure 1D** further explores the categories of AV block.

## Discussion

### Summary of results

Our data demonstrate that patients with SND do not, on average, have as high an RVP burden as patients with conduction system disease. This is particularly true for patients with a P–R interval of <250 ms. Although the current guidelines recommend programming approaches to minimize RVP in SND, this becomes difficult with more prolonged P–R intervals, and this is reflected in our data.

Although there appears to be little difference in the pacing burdens of patients paced for Mobitz 1 versus Mobitz 2 versus complete heart block, patients who present with evidence of intermittent block were paced less than those with continuous block.

In most groups, the pacing burden did not change significantly with time **(Table 2)**. In patients with SND and a P–R interval of >250 ms, there was a correlation between RVP burden and time, with an *R*^2^ value of 0.62 (*P* < .01). However, this group tended to have an RVP burden from the outset, suggesting that they may have benefitted from conduction system pacing at the time of implant.

### Significance of results

The results reiterate a number of key issues. Although the conduction system pacing indications in the European and American guidelines do not specifically include patients with SND, SND patients with a pre-implant P–R interval of >250 ms are susceptible to an elevated burden of RVP from the outset, and may, therefore, be at risk of PICM.

A more detailed assessment of the P–R interval suggests that it may have use as a predictor for switching from a low RVP burden (defined as <40%, based on the American pacing guidelines) to a higher RVP burden. In the whole bradycardia population in sinus rhythm, a 1-ms increase in the P–R interval was associated with a 10% increase in RVP burden (OR, 1.005; 95% confidence interval [CI], 1.001–1.008; *P* = .013). When only the SND population is examined, the odds ratio is 1.018 (95% CI, 1.010–1.026; *P* < .001) per 1-ms increase.

The fact that the pacing burden does not generally change very much means that RVP at any given time point during the life of a pacemaker is suitable for guiding decisions regarding device choice, eg, at the time of generator replacement.

While patients with intermittent block have a lower RVP burden, this did not rise with time during the study period. As such this would not support an idea of progression of the underlying conduction disease with time following pacemaker implant.

### Study limitations

**Figure 2:**
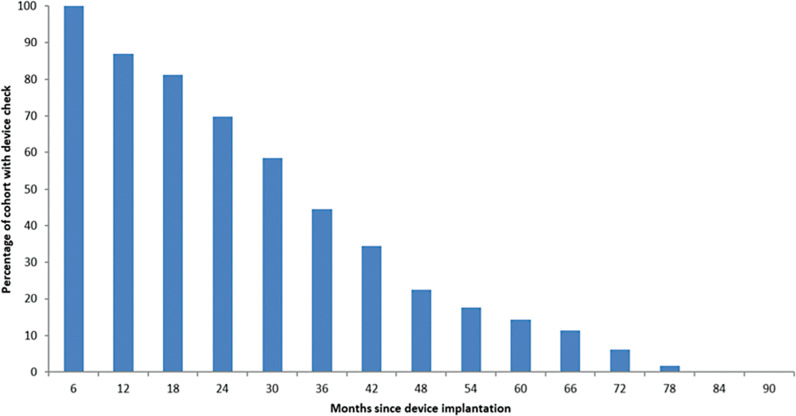
A cumulative bar chart showing the proportion of patients in the cohort with a device check at each time point. The skew reflects the proportion of new device implants as well as loss to follow-up due to relocation or death.

This was a single-center, retrospective, observational study, albeit with a relatively generalizable bradycardia- pacing population. The major limitation of this study is the relatively short median follow-up time of 826 days. As can be seen in **Figure 2**, there is significant data skew, reflecting the number of new devices that were implanted and loss of device follow-up due to patients moving away from the area or death. Given that the average battery life for a single- or dual-chamber device is now approximately a decade, a gap remains in our understanding of the temporal behavior of RVP burden in the later life of a device.

## Conclusion

Our data demonstrate that RVP burdens remain relatively stable throughout the initial years following a pacemaker implant and that presentation with intermittent AV block is associated with a lower RVP burden. Despite modern device algorithms and programming to minimize ventricular pacing, the RVP burden exceeded guideline-recommended limits for all patient groups other than those with SND and a P–R interval of <250 ms. With growing interest in conduction system pacing and guidelines now specifying RVP burden levels for recommending this approach, these results will help ensure the most appropriate devices are selected for individuals.

## Figures and Tables

**Table 1:** Baseline Demographics

	Mean (±SD) or n (%) n = 1263
Male (%)	801 (63.4)
Age (years)	78.5 ± 10.75
Indication	
SND	349 (27.6)
CSD	888 (70.3)
Other	26 (2.1)
AVB	520 (41.2)
Mobitz 1	32 (6.2)
Mobitz 2	157 (30.2)
CHB	331 (63.7)
Continuous AVB^a^	284 (54.6)
Intermittent AVB^a^	217 (41.7)
Pre-implant ECG	
AF	260 (20.6)
LBBB	158 (12.5)
RBBB	366 (29.0)
LAFB	313 (24.8)
LPFB	42 (3.3)
Pre-QRS	114.9 ± 29.5
Pre-P–R interval	216.2 ± 70.6

*Abbreviations:* AF, atrial fibrillation; AVB, atrioventricular block; CHB, complete heart block; CSD, conduction system disease; ECG, electrocardiogram; LAFB, left anterior fascicular block; LBBB, left bundle branch block; LPFB, left posterior fascicular block; RBBB, right bundle branch block; SD, standard deviation; SND, sinus node disease. ^a^Data on intermittent versus continuous block were not available for 19 patients.

**Table 2:** Correlation with Time for Different Bradycardia Populations

Population	*R* ^2^	*P* Value
SND	0.00	.86
CSD	0.28	.07
Intermittent AV block	0.22	.11
Continuous AV block	0.50	.01*
Mobitz 1	0.44	.07
Mobitz 2	0.05	.52
CHB	0.38	.02*
SND + P–R interval < 250 ms	0.00	.90
SND + P–R interval > 250 ms	0.62	<.01*

*Abbreviations:* AV, atrioventricular; CHB, complete heart block; CSD, conduction system disease; SND, sinus node disease. *Statistically significant (*P* < .05).
